# Supplementary material for: RNAi-Mediated Silencing of Laccase 2 in Culex pipiens Pupae via Dehydration and Soaking Results in Multiple Defects in Cuticular Development
Source: Insects. 2024 Mar 14;15(3):193. doi: 10.3390/insects15030193 (PMC10971320; doi:10.3390/insects15030193)
Supplement: Supplementary file 1 [file insects-15-00193-s001.zip › insects-2868833-supplementary.pdf]

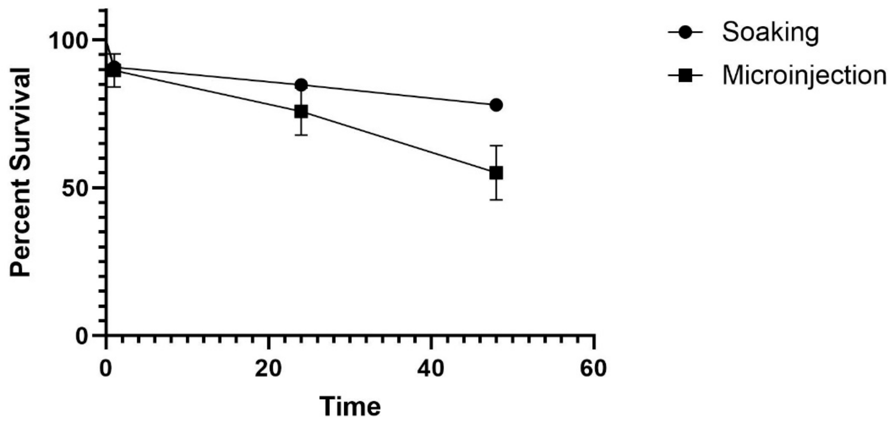

Figure S1. Survival of pupae following soaking and microinjections (χ^2=7.700, df=1, p-value=0.0055*). Percent survival pooled across all individuals exposed to each treatment type is shown. Error bars represent ± 1 SEM.
